# Decrease in self-esteem mediates the association between symptoms of social phobia and depression in middle adolescence in a sex-specific manner: a 2-year follow-up of a prospective population cohort study

**DOI:** 10.1186/1471-244X-14-79

**Published:** 2014-03-19

**Authors:** Juha-Matti Väänänen, Rasmus Isomaa, Riittakerttu Kaltiala-Heino, Sari Fröjd, Mika Helminen, Mauri Marttunen

**Affiliations:** 1 Department of Adolescent Psychiatry, Tampere University Hospital, Box 2000, Tampere 33521, Finland; 2 Tampere University Hospital, Tampere, Finland; 3 Department of Adolescent Psychiatry, University of Tampere, Medical School, Tampere University Hospital, Tampere, Finland; 4 School of Health Sciences, University of Tampere, Tampere, Finland; 5 Science Center, Pirkanmaa Hospital District and School of Health Sciences, University of Tampere, Tampere, Finland; 6 University of Helsinki, Helsinki, Finland; 7 Department of Adolescent Psychiatry, Department of Mental Health and Substance Use Services, Helsinki University Central Hospital, National Institute for Health and Welfare, Helsinki, Finland

**Keywords:** Social phobia, Depression, Adolescence, Self-esteem, Risk factor, Sex differences, Anxiety

## Abstract

**Background:**

Social phobia and depression are common, highly comorbid disorders in middle adolescence. The mechanism underlying this comorbidity, however, is unclear. Decrease in self-esteem caused by the initial disorder might play a decisive role in the development of the subsequent disorder. The present study aimed to determine whether the association between symptoms of social phobia and depression is mediated by decrease in self-esteem in mid-adolescent girls and boys.

**Methods:**

As a part of the prospective Adolescent Mental Health Cohort (AMCH), subjects of this study were 9th grade pupils (mean age, 15.5) responding to a survey conducted in 2002–2003 (T1) and to a 2-year follow-up survey in 2004–2005 (T2) (N = 2070, mean age 17.6 years, 54.5% girls).

**Results:**

Symptoms of social phobia without symptoms of depression at age 15 and symptoms of depression at age 17 were associated only among boys, and this association was mediated by decrease in self-esteem. Symptoms of depression without symptoms of social phobia at age 15 and symptoms of social phobia at age 17 were associated only among girls, and this association was partially mediated by decrease in self-esteem.

**Conclusions:**

Decrease in self-esteem plays a decisive role in the association between social phobia and depression. Self-esteem should be a key focus in interventions for adolescents suffering from social phobia or depression. Efficient intervention for the first disorder might help to prevent the decline in self-esteem and thus the incidence of the subsequent disorder. These findings are based on a sample of Finnish adolescents and should be confirmed in other jurisdictions or in more ethnically diverse samples.

## Background

Social phobia and depression are common disorders among adolescents [1-4]. These disorders are also highly comorbid with each other [3-5]. Social phobia may cause psychologic and functional deterioration leading to depression [6]. The two disorders may share underlying risk factors [7]. Further, anxiety and depression could be different manifestations of the same disorder across the course of this condition (e.g., anxiety is prominent in the early phases, and depression is prominent in later stages) [6,7].

Decrease in self-esteem is a possible indicator of psychosocial deterioration due to social phobia leading to subsequent depression, or, due to depression leading to subsequent social phobia. According to Rosenberg (1965), self-esteem is the direction of self-attitude, a favorable or unfavorable opinion of oneself. High self-esteem is the feeling that one is good enough. An individual with high self-esteem respects him/herself, and considers him/herself worthy [8].

Many studies have reported an association between low self-esteem and psychiatric disorders, especially depression, in adolescence [8-10]. Low self-esteem in adolescence predicts, and is a risk factor for, depression [11,12], while high self-esteem may protect against depression [13,14]. Rosenberg’s study in 1965 revealed that self-esteem is associated with anxiety disorders, and based on studies on the cognitive theories of social phobia [15], low self-esteem likely has an important role in adolescents suffering from social phobia. To the best of the authors’ knowledge, however, there are no adolescent population follow-up studies on low self-esteem as a risk factor for subsequent social phobia. A study among young adults showed that low self-esteem correlates with social phobia [16].

Depression among adolescents [17], and social phobia among young adults [16], and adults [18] is cross-sectionally associated with low self-esteem. Thus decrease in self-esteem might be a mediating factor between earlier social phobia and subsequent depression, or between earlier depression and subsequent social phobia. The mediating role of self-esteem, as far as the authors know, has not yet been studied.

There are sex differences in depression, social phobia, and self-esteem. Depression is more common among boys in childhood, but it is more prevalent among girls in adolescence. Female sex has also been reported to be a risk factor for depression [19,20] and its consequences [21,22]. The prevalence of social phobia is higher among girls than boys [4,23,24]. Among girls, comorbid depression emerges within a shorter interval after preceding social phobia than among boys [5]. In an earlier analysis of the present sample, social phobia predicted depression among boys, while depression predicted social phobia among girls [25]. Self-esteem is lower among girls than boys [26,27], and while self-esteem increases among boys during adolescence, the level of self-esteem throughout adolescence is not consistent among girls [27]. Some studies suggest that low self-esteem is a risk factor for depression among girls, but not among boys [12,22], while another indicates that low self-esteem is a strong risk factor for depression for both sexes [11].

### Aims of the present study

Low self-esteem is associated with social phobia and depression during adolescence. The role of low self-esteem in the development of the comorbidity between social phobia and depression, however, remains unclear. Prospective population studies among adolescents on the role of low self-esteem in the association between social phobia and depression are lacking. The main aim of the present adolescent cohort study was to examine whether the association between symptoms of social phobia and subsequent symptoms of depression or vice versa are mediated by decrease in self-esteem. Other aims of this study were to cross-sectionally examine how self-esteem is associated with symptoms of social phobia, symptoms of depression, and comorbid symptoms of social phobia and depression in adolescence, and to study sex differences in adolescent self-esteem and in the interplay between self-esteem, symptoms of depression, and symptoms of social phobia. We expected that 1) the associations between symptoms of social phobia and subsequent symptoms of depression among boys, and between symptoms of depression and subsequent symptoms of social phobia among girls would be mediated by decrease in self-esteem; 2) that adolescents with symptoms of social phobia, symptoms of depression, or comorbid disorders would have lower self-esteem than those without these symptoms; and 3) that boys would have higher self-esteem than girls.

## Methods

### Study sample and procedures

This study is part of an ongoing prospective cohort study, the Adolescent Mental Health Cohort (AMHC) study, conducted in two Finnish cities, Tampere (population 200,000) and Vantaa (population 180,000). The data collection, procedures, and measures of the study are reported in detail elsewhere [28,29]. Briefly, at the time of the baseline survey (T1), data were collected from a survey completed by ninth graders during school under the supervision of a teacher in all Finnish-speaking secondary schools in the two cities. For students absent from school that day, another opportunity to participate was offered in the school within a couple of weeks. If a student was not present on either occasion, the questionnaires were sent twice to the student by post. If no reply was received, the student was concluded to be unwilling to participate. Eligible survey participants at the 2-year follow-up (T2) were students who had participated at T1. Multiple approaches were used to contact the adolescents at follow-up. School-based surveys like that at T1 were organized in upper secondary schools and vocational schools. Adolescents not reached through schools were contacted by postal survey. Finally, the same survey was offered via the Internet to those who had not responded via their schools or by post.

The subjects of the present study were students responding to a survey conducted in 2002–2003 (T1) and at a 2-year follow-up in 2004–2005 (T2). The baseline sample comprised 1609 girls and 1669 boys with a mean ± sd age of 15.5 ± 0.39 years. Of the respondents, 69% were living in two-parent families. The distribution of the subjects’ parents’ highest education was (father/mother): 16%/13% comprehensive school only, 40%/30% primary school and vocational school, 17%/31% high school with or without vocational school, and 28%/26% academic degree [28].

A total of 2070 adolescents completed the survey at T1 and at T2, and 54.5% of the respondents who completed both surveys were girls. Mean ± sd age at T2 was 17.6 ± 0.4 years. Subjects not answering all the questions on symptoms of social phobia or depression (N = 32) were excluded from the analyses, and the final sample comprised 2038 subjects, 1154 girls and 884 boys. Sample sizes varied somewhat in the different analyses depending on baseline defaults (subsamples of being free of both SP and DEP at T1, or being free of one or the other of these disorders at T1) of each analysis.

This study was approved by the Pirkanmaa Hospital District Ethics committee, and the Tampere and Vantaa Research Approval Boards. The Finnish legislation on medical research allows subjects of age 15 years and older to provide consent. Although parental consent for participation was not required, the parents of the subjects were informed in advance by a letter.

### Drop-out

The response rate at T2 was 63.1% (2070/3278) of the baseline sample. Compared to responders of both surveys, non-responders at T2 were more likely to be boys (63.4% vs. girls 36.6%; *p* <0.001) and more likely to have symptoms of depression (11.7% vs. 9.1%, p = 0.020). Attrition was not associated with symptoms of social phobia (65.1% vs. 63.1%, p = 0.523) or self-esteem (mean 29.68 vs. 29.61, p = 0.710). Both parents’ educational level was more often comprehensive school only or lower among those who did not answer at T2 (father 18.9% vs. 15.1%, p = 0.005%, mother 16.1% vs. 12.2%, p = 0.002). Not living with both biologic parents was also more common among drop-outs (35.2% vs. 27.5%, p < 0.001).

### Measures

#### Symptoms of depression

We used the Finnish modification of the 13-item Beck Depression Inventory (RBDI) [30] to assess symptoms of depression (DEP) [31,32]. The RBDI is a widely used self-report scale measuring the severity of depressive symptoms, and its reliability and validity are good [33]. A cut-off point of 8 predicts a diagnosis of depression by a structured interview (SCAN) with good sensitivity (0.93) and specificity (0.88) [34]. The RBDI is an appropriate method for measuring depression in Finnish adolescents in population studies [30,35]. Each item is scored from 0–3 according to the severity of the symptom. Sum scores of 13 items (range 0–39) were dichotomized to non/mild depression (scores 0–7) and moderate/severe depression (scores 8–39) [31].

#### Symptoms of social phobia

The Social Phobia Inventory (SPIN) [36], a 17-item self-report questionnaire on a 5-point Likert-type scale, was used to measure symptoms of social phobia (SP). The scale was demonstrated to have good reliability and construct validity when used among English-speaking and Finnish adolescents [37,38]. Sum scores of the SPIN were used to identify participants with SP. For the Finnish translation of the SPIN, a cut-off point of 24 has 81.8% sensitivity and 85.1% specificity for a diagnosis of SP [38], and this cut-off point was used in the present study.

#### Self-esteem

Self-esteem (SE) was measured using the Finnish translation of the Rosenberg Self-Esteem Scale (RSES) [8]. The RSES is a 10-item scale that measures self-esteem, with 5 items reflecting high self-esteem and 5 reflecting low SE. In each item, participants were asked to indicate their agreement to statements on a four-point scale (1 = strongly agree to 4 = strongly disagree). We scored the answers from 1 to 4 on each item, scoring a total sum from 10 to 40; the higher the sum score, the higher the SE. We used continuous sum scores of the RSES in our analysis. The RSES was originally tested on a large sample of high-school students and found to have good face validity in an adolescent population [8]. Since then, the RSES has been widely used in adolescent studies in many countries [11,27].

### Missing values

Cases were excluded if more than three items of our measures were unanswered. If three or fewer items were unanswered, missing values were replaced with the mean value of the subject’s responses to the other items of the scale.

### Covariates

The covariates controlled for in the statistical analyses were age, family structure (living with both biologic parents/living in some other family structure), both parents’ highest educational qualification (comprehensive school only/higher education), and externalizing symptoms, measured by the Youth Self Report [39], at T1. The externalizing scale of the Youth Self Report as a continuous sum score was used in the present study. These covariates were selected because earlier studies suggested that these covariates impact the main variables of interest in the present study [8,13,20,40,41].

### Statistical analyses

#### Group comparisons

The study sample was divided into four groups according to disorder status: adolescents without symptoms of social phobia or symptoms of depression (no-SP/DEP; SPIN score <24, R-BDI score <8), with symptoms of social phobia and without symptoms of depression (SP; SPIN ≥24, R-BDI <8), with symptoms of depression and without symptoms of social phobia (DEP; SPIN <24, R-BDI ≥8), and with both symptoms of social phobia and symptoms of depression (comorbid-SP/DEP; SPIN ≥24, R-BDI ≥8). Descriptive statistics are provided for the RSES sum score according to current SP, DEP, or comorbid-SP/DEP at T1 and T2 for the whole sample and separately for girls and boys.

#### Mediation

Baron and Kenny (1986) pointed out that an association between two variables (a) and (b) is mediated by a third variable (c) if 1) there is an association between variable (a) at T1 and variable (b) at T2, 2) variable (a) at T1 is associated with a third variable (c), 3) the third variable (c) is associated with variable (b) at T2, and 4) the association between variables (a) at T1 and (b) at T2 disappears after adding the variable (c) into the model, and these authors called this a perfect mediation. If the association between variables (a) at T1 and (b) at T2 persists but weakens, it is called a partial mediation [42].

#### Decrease in SE as a mediating factor

We examined whether decreased SE is a mediating factor of the association of SP at T1 to DEP with or without SP at T2 (N = 1852, girls n = 1021, boys n = 831), and DEP at T1 to SP with or without DEP at T2 (N = 1854, girls n = 1038, boys n = 816) by logistic regression analyses as described by Baron and Kenny (1986). We named the proposed mediating factor ‘change in SE from T1 to T2’, that is [SE at T2 – SE at T1]. If SE increased from T1 to T2, ‘change in SE from T1 to T2’ would be positive, and if SE decreases, it would be negative. We evaluated the impact of SP or DEP at T1 on ‘change in self esteem between T1 and T2’ by binary logistic regression analysis. Next, we added, ‘change in SE from T1 to T2’, and covariates, to binary logistic regressions. Two logistic regression analyses were performed. In the first, SP at T1 was the independent variable and DEP with or without SP at T2 was the dependent variable. In the second analysis, DEP at T1 was the independent variable and SP with or without DEP was the dependent variable. To avoid the confounding effect of baseline SE, we controlled SE at T1 in our model. We performed a logistic regression analysis to determine the association between SE at T1 and SP or DEP at T2 in a subsample of no-SP/DEP at T1.

In the logistic regression analyses, odds ratio (OR) with 95% confidence interval (CI) was used to show associations between independent and dependent variables of the model. A p-value of less than 0.05 was considered to indicate statistical significance. Statistical significance tests were two-tailed.

The data analyses were performed using SPSS, version 16.0 (SPSS Inc., Chicago, IL, USA).

## Results

### Self-esteem according to symptoms of depression and symptoms of social phobia

Boys had higher RSES scores than girls at ages 15 and 17 in the whole sample and in the no-SP/DEP group. In addition, the self-esteem of boys was significantly higher than that of girls at age 15 in the SP group and at age 17 in the comorbid-SP/DEP group. The RSES scores were significantly lower in all disorder groups compared to the no-SP/DEP group at both age 15 and 17 in both sexes (Table 1). When the disorder groups were compared, current SE was highest in the SP group in both sexes at both ages. Among girls, SE was also lower in the comorbid-SP/DEP than in the DEP group (Table 1).

**Table 1:** Means of Rosenberg Self-Esteem Scale at 15 and at 17, among boys and girls

		**15 y**				**17 y**			
		**All**	**Boys**	**Girls**	**p**^**(2**^	**All**	**Boys**	**Girls**	**p**^**(2**^
Current disorder status at 15 or at 17	No-SP/ DEP	30.7	31.4	30.1	<0.001^(2^	31.6	32.3	31.1	<0.001^(2^
		N = 1723	N = 775	N = 948		N = 1720	N = 767	N = 953	
	SP	27.3	28.6***^(1^	26.1***^(1^	=0.004^(2^	27.2	27.6***^(1^	26.9***^(1^	ns^(2^
		N = 108	N = 50	N = 58		N = 129	N = 48	N = 81	
	DEP	23.0	23.5***^(1^	22.7***^(1^	ns^(2^	23.0	22.5***^(1^	23.2***^(1^	ns^(2^
		N = 107	N = 35	N = 72		N = 80	N = 23	N = 57	
	Com-SP/ DEP	19.8	20.6***^(1^	19.6***^(1^	ns^(2^	20.9	22.6***^(1^	19.9***^(1^	=0.006^(2^
		N = 78	N = 18	N = 60		N = 96	N = 34	N = 62	
	All	29.7	30.8	28.9	<0.001^(2^	30.5	31.4	29.8	<0.001^(2^
		N = 2016	N = 878	N = 1138		N = 2025	N = 872	N = 1153	

no-SP/DEP = no symptoms of social phobia or depression (SPIN score <24, R-BDI score <8), SP = symptoms of social phobia without symptoms of depression (SPIN ≥24, R-BDI <8), DEP = symptoms of depression without symptoms of social phobia (SPIN <24, R-BDI ≥8) com-SP/DEP = comorbid symptoms of social phobia and depression (SPIN ≥24, R-BDI ≥8).

^(1^ = Statistical significance of differences between disorders to no SP no DEP group, Mann–Whitney –test, *** = p < 0.001.

^(2^ = Statistical significance of differences between boys and girls, Mann-Whitney–test.

### Self-esteem as a mediator of the association between symptoms of social phobia and symptoms of depression

**Figure 1:**
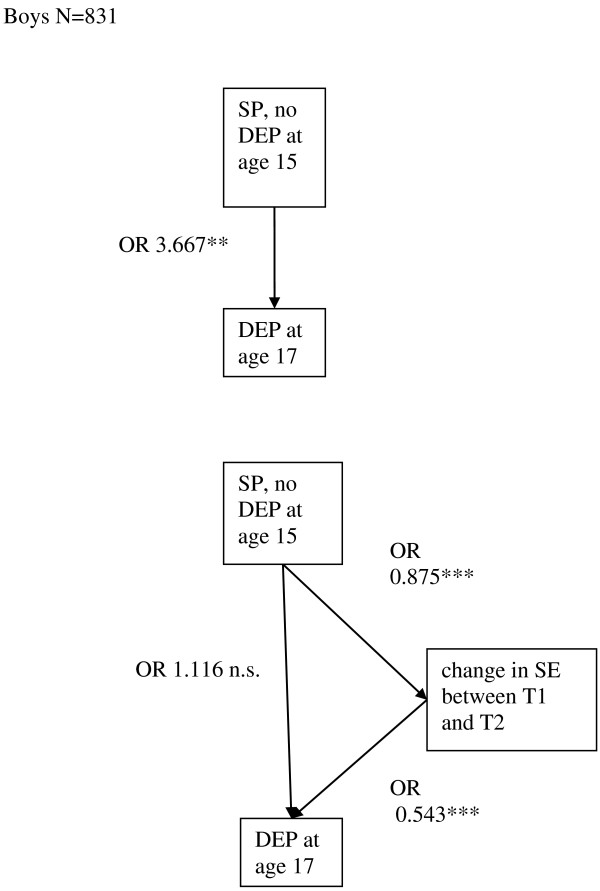
SE as a mediator between SP without DEP at T1 and DEP (with or without SP) at T2 among boys, SE=self esteem, SP=symptoms of social phobia, DEP=symptoms of depression, OR=odds ratio, ***=p<0.001, **=p≤0.01, *= p<0.05, n.s.=p>0.05.

SP at T1 was significantly associated with DEP with or without SP at T2 only among boys (boys, n = 831, OR = 3.67, 95% CI = 1.54-8.76, p = 0.003, girls n = 1021, OR = 2.11, 95% CI = 0.85-5.14, ns). SP at T1 was associated with ‘change in SE from T1 to T2’, and ‘change in SE from T1 to T2’ was associated with DEP with or without SP at T2. The addition of ‘change in SE from T1 to T2’ into the model, made the association of SP at T1 with DEP with or without SP at T2 non-significant. Thus, the association of SP at T1 with DEP with or without SP at T2 among boys was mediated by ‘change in SE from T1 to T2’ when controlling for SE at T1 (Figure 1).

**Figure 2:**
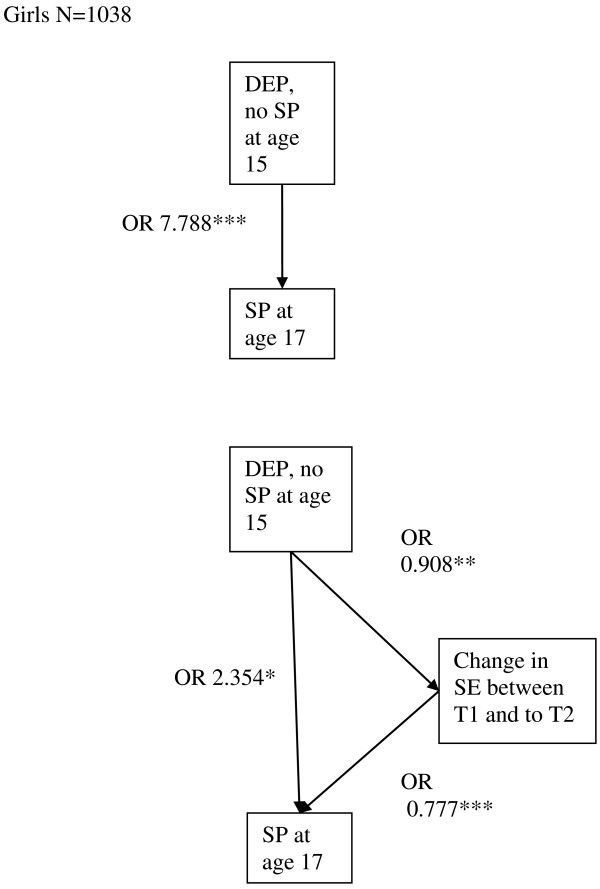
SE as a partial mediator of the association between DEP without SP at T1 and SP at T2 among girls, SE=self esteem, SP= symptoms of social phobia, DEP= symptoms of depression, OR=odds ratio, ***=p<0.001, **=p<0.01, *= p<0.05, n.s.=p>0.05.

DEP at T1 was a significant predictor of SP with or without DEP at T2 only among girls (girls, n = 1038, OR = 7.79, 95% CI = 4.53-13.39, p < 0.001, boys n = 816, OR = 2.47, 95% CI = 0.92-6.66, ns). DEP at T1 was associated with ‘change in SE from T1 to T2’, and ‘change in SE from T1 to T2’ was associated with SP with or without DEP at T2. Although adding ‘change in SE from T1 to T2’ affected the OR of the association between DEP at T1 and SP with or without DEP at T2, the association remained statistically significant. Thus, among girls, the association between DEP without SP at T1 and SP at T2 was partially mediated by the ‘change in SE from T1 to T2’ when controlling for SE at T1 (Figure 2).

Among girls, there was a significant association between DEP at T1 and ‘change in SE between T1 and T2’ (Figure 2), while there was no statistically significant association between SP at T1 and ‘change in SE from T1 to T2’ when SE at T1 was controlled for (OR = 0.96, 95% CI = 0.90-1.02, ns). Among boys, SP at T1 was associated with ‘change in SE between T1 and T2’ (Figure 1), whereas DEP at T1 was not associated with ‘change in SE from T1 to T2’ (OR = 0.97, 95% CI 0.89-1.04, ns) when controlling for SE at T1.

Among boys in the no-SP/DEP group, SE at T1 was not associated with DEP at T2 in the model with all covariates (OR = 0.92, 95% CI 0.84-1.01, ns). Among girls, SE at T1 was significantly associated with SP at T2 after adding covariates to the analysis (OR 0.91, 95% CI 0.85- 0.98, p = 0.01) in the no-SP/DEP group at T1.

## Discussion

Our main hypothesis that decrease in self-esteem mediates the associations of earlier symptoms of social phobia without symptoms of depression and subsequent symptoms of depression (boys), or symptoms of depression without symptoms of social phobia and subsequent symptoms of social phobia (girls) was supported. Among boys, the association between symptoms of social phobia without symptoms of depression at 15 and symptoms of depression at 17 was mediated by decrease in self-esteem. This mediation was perfect based on the definition of Baron and Kelly (1986). Among girls, the association between symptoms of depression without symptoms of social phobia and subsequent symptoms of social phobia was partially mediated by decrease in self-esteem.

To the best of our knowledge, the mediating role of decrease in self-esteem in the associations between social phobia and depression in adolescence has not been investigated. Although decrease in self-esteem emerged only as a partial mediator between symptoms of depression and subsequent symptoms of social phobia among girls, this finding is noteworthy. As Baron and Kelly (1986) noted, “in psychological research a perfect mediation is a rare phenomena, but also in partial mediation, a significant reduction in the association between independent and dependent variable, demonstrates that a given mediator indeed is a potent, albeit not both necessary and sufficient condition for an effect to occur”.

Our findings are in accordance with sociometric theory that self-esteem is affected by the degree to which a person is included/accepted or excluded/rejected by others [43]. In both social phobia and depression, there is perceived exclusion or rejection by others. Perceived acceptance or rejection by others affects self-esteem especially strongly during adolescence [43]. Decrease in self-esteem during adolescence further leads to the development of a subsequent disorder (depression or social phobia).

Dumont and Provost (1999) reported that low self-esteem is associated with avoidance as a coping style and high self-esteem is associated with active problem-solving as a coping style. Thus, they speculated that the association between self-esteem and depression is mediated by the coping style. As avoidance is one of the main characteristics of social phobia, this association might also be true in our study, and the role of the coping style in the associations detected in our study warrants further study [44].

In cross-sectional analyses, low self-esteem at ages 15 and 17 was associated with both symptoms of depression and social phobia, as we expected. The association was strongest in comorbid disorders and weakest in symptoms of social phobia without symptoms of depression. These findings are consistent with those of earlier studies on social phobia in adult populations [16,18] and with adolescent studies on depression [8,10]. To the best of our knowledge, however, there are no earlier studies comparing the strengths of the associations of self–esteem and social phobia, depression, or comorbid social phobia and depression among boys and girls.

As we expected based on earlier studies, boys had higher self-esteem than girls if they had no symptoms of either social phobia or depression [27]. This did not hold true, however, for those suffering from symptoms of depression without symptoms of social phobia at ages 15 or 17, comorbid symptoms of depression and symptoms of social phobia at age 15, and symptoms of social phobia without symptoms of depression at age 17. Thus, these disorders seemed to be more strongly associated with boys’ self-esteem than girls’.

The role of low self-esteem in the emergence of social phobia and depression in middle adolescence seems to differ among boys and girls. Decrease in self-esteem was a perfect mediator for the association between symptoms of social phobia and subsequent symptoms of depression among boys, and a partial mediator for the association between symptoms of depression and later symptoms of social phobia among girls. Also, symptoms of social phobia and symptoms of depression in middle adolescence differentially affected self-esteem depending on sex. As we hypothesized, symptoms of social phobia at age 15 led to a decrease in self-esteem among boys but not among girls, and symptoms of depression at 15 led to a decrease in self-esteem among girls but not among boys. These sex differences in the associations might be explained by different attributions of self-esteem among boys and girls [8].

Symptoms of social phobia and symptoms of depression differentially affect self-esteem in girls and boys. Girls may in general be more home-directed [45] and centered on one or two very good friends, while boys may orient more outward from home and be centered on larger peer groups [45]. Also, activity, action, and, for example, team sports and an active role in social situations might be more important toward building male self-esteem than female self-esteem [8]. Thus, symptoms of social phobia may have a greater effect on self-esteem among boys than girls. Depression is associated more directly with self-image, physical self-image [46], and perceived social acceptance, which could have a greater impact on self-esteem among girls than boys, leading to the deterioration of social functioning [19].

According to the results of the present study, among boys, symptoms of social phobia lead to deterioration of self-esteem, which in turn leads to subsequent symptoms of depression. This is especially likely as low self-esteem in the absence of symptoms of social phobia was not a risk factor for later symptoms of depression among boys. Among girls, this route is more complex. Symptoms of depression lead to deterioration of self-esteem among girls. Decreased self-esteem is a risk factor for symptoms of social phobia. Symptoms of depression and low self-esteem are also factors leading to symptoms of social phobia *per se,* or via other mediating factors among girls.

The present study was based on a large population sample. The coverage of compulsory comprehensive school until age 16 in Finland is greater than 99%. This cohort may thus be considered representative of the age group studied. The sample was also homogeneous regarding age. There are, however, some limitations to the current study that must be acknowledged for interpretation of our results. Firstly, while the response rate to the Adolescent Mental Health Cohort baseline survey was good, drop-out during the follow-up was relatively high, and was related to symptoms of depression at baseline. Although the baseline sample was large, the number of adolescents suffering from symptoms of depression and social phobia was quite low, and the non-significant findings in the difference in self-esteem between the different disorder groups may have been due to insufficient statistical power. This possible lack of statistical power, however, does not affect our main finding regarding the mediating effect of decrease in self-esteem. In addition, the lack of diagnostic interviews is a limitation. Adolescents are, however, able to reliably report their health in several psychological disorders [47], and the measures used in the present study were previously used successfully in large community samples of adolescents [12,30,35,38]. Further, the lack of control for other possible disorders may affect our main results. Externalizing symptoms were controlled for, however, which is rare in studies on depression and anxiety. Further, different methods used to obtain the information in the follow-up may have affected the responses of the participants. For example, students who responded to measures at home or via the internet or postal services may be more or less likely to provide accurate results than those responding in supervised school settings.

## Conclusions

Among boys, decrease in self-esteem mediates the association between earlier symptoms of social phobia and later symptoms of depression, and among girls it partially mediates the association between earlier symptoms of depression and later symptoms of social phobia. It is important to detect social phobia and depression among adolescents. This study emphasizes the importance of self-esteem as a central focus in treatment interventions for social phobia and depression among adolescents. Efficient intervention for the first disorder might help to prevent the decline in self-esteem and thus the incidence of the subsequent disorder. These findings are based on a sample of Finnish adolescents and should be confirmed in other jurisdictions or in more ethnically diverse samples.

## Abbreviations

AMHC: Adolescent Mental Health Cohort; BDI-13: 13-item Beck Depression Inventory; CI: Confidence interval; DEP: Depression; K-SADS-PL: Schedules for affective disorders and schizophrenia for school-aged children –lifetime version; OR: Odds ratio; RSES: Rosenberg’s Self Esteem Scale; SCAN: Schedules for clinical assessment in neuropsychiatry; SD: Standard deviation; SE: Self-esteem; SP: Social phobia; SPIN: Social Phobia Inventory; SPSS: Statistical package for the social sciences; T1: Time of the baseline survey; T2: Time of the 2-year follow-up survey; YSR: Youth Self Report.

## Competing interests

The authors declare that they have no competing interests.

## Authors’ contributions

J-MV designed the study, performed the data and statistical analyses, interpreted the results, and wrote the manuscript. SF, MM, and RK-H designed the Adolescent Mental Health Cohort Study, organized the data collection of the Adolescent Mental Health Cohort Study, and participated in planning the design of the data analyses and in writing the manuscript. RI participated in planning the design of the data analyses and in writing the manuscript. MH participated in planning the design of the data analyses, interpreting the data, in planning and interpreting the statistical analyses, and in writing the manuscript. All authors read and approved the final manuscript.

## Pre-publication history

The pre-publication history for this paper can be accessed here:

http://www.biomedcentral.com/1471-244X/14/79/prepub
